# Association between Metabolic Phenotypes of Body Fatness and Incident Stroke: A Prospective Cohort Study of Chinese Community Residents

**DOI:** 10.3390/nu14245258

**Published:** 2022-12-09

**Authors:** Minhua Tang, Qi Zhao, Kangqi Yi, Yiling Wu, Yu Xiang, Maryam Zaid, Shuheng Cui, Xuyan Su, Yuting Yu, Genming Zhao, Yonggen Jiang

**Affiliations:** 1 Key Laboratory of Public Health Safety of Ministry of Education, Department of Epidemiology, School of Public Health, Fudan University, Shanghai 200032, China; 2 Songjiang District Center for Disease Control and Prevention, Shanghai 201600, China

**Keywords:** obesity phenotypes, stroke, body mass index, waist circumference, cohort study

## Abstract

This study aimed to assess the association of body mass index (BMI)-based and waist circumference (WC)-based metabolic phenotypes with the risk of stroke among Chinese community residents. A total of 34,294 participants (mean ± standard deviation age: 56.05 ± 11.26 years) with no previous stroke diagnosis history were included in this cohort study. BMI-based metabolic phenotypes were classified into eight groups: metabolically healthy and normal weight (MHNW), metabolically healthy and underweight (MHUW), metabolically healthy and overweight (MHOW), metabolically healthy and obese (MHO), metabolically unhealthy and normal weight (MUNW), metabolically unhealthy and underweight (MUUW), metabolically unhealthy and overweight (MUOW), and metabolically unhealthy and obese (MUO). WC-based metabolic phenotypes were classified into four groups: metabolically healthy and normal WC (MHNWC), metabolically healthy and oversized WC (MHOWC), metabolically unhealthy and normal WC (MUNWC), and metabolically unhealthy and oversized WC (MUOWC). The association of these phenotypes with developing stroke events was examined using proportional hazards models. A total of 546 cases of first-stroke onset were recorded over a median follow-up time of 4.97 years. Compared with the reference group, the obesity phenotypes showed higher risks for stroke. The adjusted HRs (95% CIs) of MHUW, MHOW, MHO, MUNW, MUUW, MUOW, and MUO phenotypes were 1.01 (0.41, 2.49), 1.47 (1.09, 2.00), 1.33 (0.80, 2.22), 2.49 (1.87, 3.30), 3.92 (1.44, 10.72), 2.14 (1.64, 2.79), and 2.60 (1.91, 3.55), respectively. The adjusted HRs (95% CIs) of MHOWC, MUNWC, and MUOWC were 1.41 (1.02, 1.94), 2.25 (1.76, 2.87), and 2.16 (1.63, 2.87), respectively. The metabolic phenotypes defined by an alternative definition all showed significant positive associations (except for MHUW), with the adjusted HR ranging from 1.51 to 3.08 based on BMI and from 1.68 to 2.24 based on WC. The risk of stroke increased with the increase in metabolic abnormality numbers in different BMI and WC groups (all *p* trend < 0.001). The present study suggests that maintaining normal body weight or WC and improving metabolic health are of great significance in preventing cerebrovascular diseases.

## 1. Introduction

For Chinese adults, stroke is now the leading cause of disability and mortality [1], accounting for 2.19 million deaths and 45.9 million DALYs due to stroke events occurring in 2019 [2]. According to the Global Burden of Disease report (data from 2016), the lifetime risk of stroke occurring in individuals over 25 years old was 24.9% globally, and China had the highest risk, reaching 39.3% (41.1% for males and 36.7% for females) [3]. Hypertension, obesity, diabetes, and dyslipidemia are known independent risk factors for stroke, and the number of people suffering from these chronic diseases continues to rise in our country, which will lead to a further increase in the mortality and morbidity of cardiovascular diseases (CVDs) [4]. Therefore, great challenges exist in the prevention and control of stroke events.

In China, overweight and obesity have become essential public health issues, affecting approximately 50% (overweight prevalence: 34.3% and obesity prevalence 16.4%) of adults [5]. Obesity has proven to be related to metabolic diseases, such as hypertension [6], diabetes [7], and dyslipidemia [8], and an increased risk of stroke events has been presented in previous studies [9,10]. Except for general obesity assessed by body mass index (BMI) measurements, central obesity measured by waist circumference (WC) was considered to be more closely related to CVD events [11]. However, not all obese individuals are accompanied by those metabolic abnormalities. The metabolically healthy and obese (MHO) or overweight (MHOW) phenotype is characterized by high BMI levels and insulin sensitivity but is free of high levels of blood pressure or serum lipids [12]. MHO or MHOW was reported to be associated with higher CVD risks [13,14,15,16], but some studies demonstrated that MHO or MHOW is a healthy phenotype [17,18,19,20]. Therefore, the role of the MHO or MHOW phenotype in stroke requires further studies. The risk of stroke might vary in metabolic phenotypes of different categories of body fatness. However, research so far has focused on the association between MHO and stroke risks but ignored individuals with underweight or central obesity. To address the limitation of earlier studies, the study aims to comprehensively evaluate the stroke risks in different metabolic phenotypes grouped by BMI and WC measurements based on an ongoing cohort study conducted in Shanghai, China.

## 2. Materials and Methods

### 2.1. Study Design and Participants

The present study was part of the Shanghai Suburban Adult Cohort and Biobank (SSACB) study. From June 2016 to December 2017, the baseline survey was performed in four communities in Songjiang district, Shanghai (two urban areas: Sheshan and Zhongshan; two non-urban areas: Maogang and Xinqiao). A stratified multistage random sampling method was conducted to recruit the subjects. The details of the study design have been described previously [21]. Briefly, in the first stage, the four aforementioned areas were selected based on the geographical characteristics, economic status, and population distribution of Songjiang District. In the second stage, one-third of the committees or villages were randomly selected from each community. In the third stage, all residents aged 20–74 years who had lived in the communities for at least five years were invited to the cohort. The participation rate was 90%, and 10% of people did not want to participate. A total of 36,404 participants were enrolled and completed the questionnaires, anthropometric measurements, and biochemical tests. Overall, 1129 participants who were previously diagnosed with stroke were excluded from the study. Furthermore, the study also excluded the participants with missing data for height, weight, SBP, DBP, serum lipids, FPG, and HbA1c (*n* = 981). Finally, 34,294 participants were deemed eligible and included in this analysis. Every participant was linked to their diagnosis and medication-use records in the local health information database, including the CVD reporting data, the cause-of-death surveillance data, and electronic medical record (EMR) data. Multiple database links were accessed by unique identification card number matching. All subjects signed a written informed consent form prior to the research. The ethical review of this study was approved by the Medical Research Ethics Committee of the School of Public Health, Fudan University (IRB#2016-04-0586).

### 2.2. Questionnaire Interview and Anthropometric Measurement

Demographic information (gender, age, marital status, education degree, retirement status, and place of residence), self-reported chronic disease history (stroke, diabetes, and hypertension), behavioral lifestyles (physical activities, smoking, and alcohol drinking), and dietary intake (fruit, vegetables, fish, and processed and unprocessed meats) were collected by well-trained working staff through face-to-face interviews. The smoking index was calculated by multiplying packets per day by the smoking years and was divided into four groups (packet-year): non-smoker, <20.0, 20.0–39.9, and ≥40.0. Alcohol drinking status was categorized into three groups: never, former, and current. The metabolic equivalent task (MET) multiplied by the total weekly minute count was used to evaluate physical activity [22] and was categorized into three groups (MET-mins/week): low (<2000), moderate (2000–5999), and high (≥6000) [23]. Dietary intake was assessed based on the frequency and the amount of food on average in the last twelve months.

When measuring the height and weight of the participants, the removal of shoes and hats and wearing lightweight clothing were required. The height and weight measurements were accurate to 0.1 cm and 0.1 kg, respectively, and the average values of the two measurements were recorded. The BMI was assessed as body weight (kilograms, kg) divided by height squared (meters^2^, m^2^). When measuring the WC, a soft ruler was used to measure the rim of the midpoint line between the lowest rib and the upper iliac crest edge under minimum-breathing conditions. The WC was measured twice with the average value being recorded and was accurate to 0.1 cm. Systolic blood pressure (SBP) and diastolic blood pressure (DBP) were measured on the upper-right arm using an electronic sphygmomanometer after five minutes of rest. The blood pressure was measured three times with the mean measurement being recorded.

### 2.3. Laboratory Measurement

Before collecting the blood samples, an empty stomach of at least 8 h was required. Serum lipids, including low-density lipoprotein cholesterol (LDL-C), triglyceride (TG), and high-density lipoprotein cholesterol (HDL-C), were tested using an automatic biochemical analyzer (Roche Cobas-C501) with enzymatic colorimetry (HDL-C and LDL-C) and colorimetry (TG) methods. Fasting plasma glucose (FPG) was tested using an automatic biochemical analyzer (Roche Modular-P800) with hexokinase methods. Glycated hemoglobin (HbA1c) was tested by the Tosoh G8 automatic glycohemoglobin analyzer using high-pressure liquid chromatography methods. These samples were all measured at the Dian Medical Laboratory Center.

### 2.4. Definitions of Metabolic Phenotypes of Body Fatness

The BMI groups were defined according to Chinese standards and divided into underweight (<18.5 kg/m^2^), normal weight (18.5–23.9 kg/m^2^), overweight (24.0–27.9 kg/m^2^), and obesity (≥28.0 kg/m^2^) [24]. An oversized WC was defined as 90 cm and larger in men and 80 cm and larger in women [25]. An unhealthy metabolic status was defined as two or more of the following four conditions: 1. BP ≥ 130/85 mmHg or undergoing anti-hypertensive medical treatment; 2. HDL-C < 1.29 mmol/L for women and <1.04 mmol/L for men, or drug treatment; 3. TG ≥ 1.7 mmol/L; and 4. FPG ≥ 5.6 mmol/L or receiving treatment for diabetes [26].

The alternative definition of abnormal metabolism was defined as two or more of the five following conditions: 1. BP ≥ 130/80 mmHg or taking hypotensive medications; 2. LDL-C ≥ 3 mmol/L or using anti-hyperlipidemic drugs; 3. TG ≥ 2.3 mmol/L; 4. HDL-C ≤ 1.0 mmol/L; and 5. HbA1c ≥ 6% or drug treatment [13]. The BMI groups were defined based on the World Health Organization (WHO) definitions [27], with different overweight and obesity cutoffs: 18.5–24.9 kg/m^2^ was normal weight, 25.0–29.9 kg/m^2^ was overweight, and ≥30.0 kg/m^2^ was obese.

The BMI-based metabolic phenotypes were classified into eight groups: metabolically healthy and normal weight (MHNW), metabolically healthy and underweight (MHUW), MHOW, MHO, metabolically unhealthy and normal weight (MUNW), metabolically unhealthy and underweight (MUUW), metabolically unhealthy and overweight (MUOW), and metabolically unhealthy and obese (MUO). The WC-based metabolic phenotypes were classified into four groups: metabolically healthy and normal WC (MHNWC), metabolically healthy and oversized WC (MHOWC), metabolically unhealthy and normal WC (MUNWC), and metabolically unhealthy and oversized WC (MUOWC).

### 2.5. Follow-Up and Outcomes

Fatal and non-fatal stroke events were documented using the CVD reporting data, the cause-of-death surveillance data, and the EMR data. The CVD reporting system records the stroke events of local residents diagnosed by clinicians at various hospitals in Shanghai and includes the names of the disease, subtypes of the disease, numbers of stroke onset (once, twice, three times...), date of onset, and date of death. The EMR system records the details of the diagnosis, including the name of a disease diagnosis, ICD-10 codes, and date of diagnosis. Stroke-related deaths were collected on the cause-of-death surveillance system combined with the CVD reporting system. The outcomes were recorded from the time participants were enrolled in the cohort until 31 December 2021, including ischemic and hemorrhage strokes. The information on the outcome collection has been described previously [28]. The 10th Revision of the International Classification of Diseases (ICD-10) codes included I60–I64.

### 2.6. Statistical Analysis

Participants’ baseline characteristics are presented as frequency and corresponding column proportions for the categorical data. The median value with the interquartile range (skewed distribution), as well as the mean value with the standard deviation (SD) (normal distribution), were described for the continuous data. The Kolmogorov–Smirnov method was used to test the normal or non-normal distribution of the data. The characteristics of the participants were described according to the BMI and WC groups. The difference between these groups was tested with the χ^2^ test for the categorical data. The one-way ANOVA analysis (BMI group), Student *t*-test (WC group), or Wilcoxon–Mann–Whitney U test (skewed distributed variables) was used to compare the continuous data. Cox regression analyses were performed to assess the association of BMI-based and WC-based metabolic phenotypes with stroke events, reporting the hazard ratio (HR) and 95% confidence interval (CI) as the results. The multivariate model was fully adjusted for age, gender, place of residence, marital status, education degree, retirement, physical activities, smoking index, alcohol drinking, and consumption of fruit, vegetables, fish, and processed and unprocessed meats. The BMI was additionally adjusted in the analysis of WC-based metabolic phenotypes. In addition, we analyzed the relationship between the number of metabolic abnormalities and stroke risks in different BMI and WC groups, with 0 metabolic abnormalities as the reference group. SAS 9.4 (SAS Institute Inc., Cary, NC, USA) software was applied to all data analyses. A statistically significant *p*-value was set at less than 0.05 (two-tailed value).

## 3. Results

The baseline characteristics are described according to the BMI (Table 1) and WC (Table S1) groups. We analyzed 34,294 participants with a mean age of 56.05 ± 11.26 years. The mean values of BMI and WC were 24.38 ± 3.35 kg/m^2^ and 81.62 ± 9.42 cm, respectively. The prevalence of overweight was 39.33%, obesity was 13.28%, and central obesity was 40.84%, respectively. Participants who were overweight and obese were associated with the female gender; older age; higher levels of TG, LDL-C, FPG, HbA1c, SBP, DBP, and WC, but less fruit intake and lower levels of education and HDL-C when compared with the normal-weight participants (*p* < 0.001). The results were similar when comparing participants with an oversized WC to normal WC (*p* < 0.001) (Table S1). Among the participants with normal weight and WC, 31.53% and 35.14% were metabolically unhealthy, respectively. The prevalence of unhealthy metabolic status and the mean number of metabolic abnormalities increased with BMI among all subjects. The proportions of BMI-based metabolic phenotypes were as follows: MHNW, 30.64%; MHUW, 2.34%; MHOW, 17.58%; MHO, 4.15%; MUNW, 14.11%; MUUW, 0.30%; MUOW, 21.75%; and MUO, 9.14%. The proportions of WC-based metabolic phenotypes were as follows: MHNWC, 38.37%; MHOWC, 16.33%; MUNWC, 20.79%; and MUOWC, 24.51%.

Over a median follow-up time of 4.97 years (a total of 166,183.17 person-years of follow-up sessions), 546 participants experienced stroke events. The incidence density of stroke was 328.55 per 100,000 person-years (95% CI: 304.04–356.07). Table 2 presents the association of metabolic phenotypes of body fatness with stroke risks. When the metabolic phenotypes were defined according to BMI, MHOW and all the metabolically unhealthy phenotypes (regardless of normal weight, underweight, overweight or obese) presented a greater risk of stroke compared with the MHNW phenotype after full adjustment. HRs and 95% CIs were 1.47 (1.09, 2.0) for MHOW, 2.49 (1.87, 3.30) for MUNW, 3.92 (1.44, 10.72) for MUUW, 2.14 (1.64, 2.79) for MUOW, and 2.60 (1.91, 3.55) for MUO phenotypes, and MUUW presented the highest HR among all the BMI-based metabolic phenotypes. MHO presented no significant relationship with stroke (1.33, 0.80–2.22). In different gender groups, MHOW was only positively related to high stroke risk among males.

Similar positive associations are presented between WC-based metabolic phenotypes and stroke in Table 2. HRs and 95% CIs were 1.41 (1.02, 1.94) for MHOWC, 2.25 (1.76, 2.87) for MUNWC, and 2.16 (1.63, 2.87) for MUOWC phenotypes. MHOWC was more significantly associated with stroke occurring amongst females rather than males (*p* < 0.05). The stroke risk of the metabolically unhealthy participants was much higher than that of metabolically healthy participants across the BMI and WC categories.

**Figure 1:**
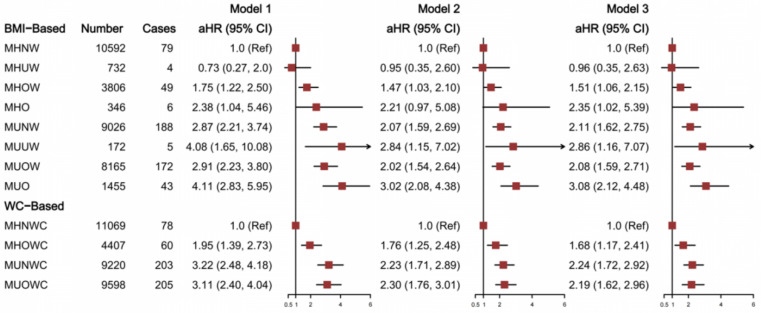
Hazard ratios for stroke according to metabolic phenotypes with an alternative definition. Model 1, unadjusted. Model 2, adjusted for gender and age. Model 3, adjusted for gender, age, education degree, marital status, retirement, place of residence, smoking index, alcohol drinking, physical activities, and consumption of fruit, vegetables, fish, and processed and unprocessed meats. BMI was additionally adjusted in the WC-based metabolic phenotypes in Model 3.

Figure 1 demonstrates the stroke risk in different metabolic phenotypes according to the alternative phenotype definitions. The prevalence of an unhealthy metabolic status was 54.88% (18,819/34,294), and MHO was 1.01% (346/34,294) among all subjects. Compared with the reference group, all the metabolic phenotypes (except for MHUW) presented a positive association with stroke risk after fully adjusting the confounders. HR (95% CI) was 2.35 (1.02, 5.39) for the MHO phenotype.

We further analyzed the relationship between the number of metabolic abnormalities and stroke risks in different BMI and WC groups (Table 3). Metabolic abnormality numbers were classified into five groups (0, 1, 2, 3, and 4). The stroke risk increased as the number of metabolic abnormalities increased in different BMI and WC groups (all *p* trend < 0.001).

## 4. Discussion

This prospective study using a large sample of 34,294 participants determined the risk of stroke in metabolic phenotypes of different categories of body fatness. The overall MHO prevalence defined by BMI was 1.0–4.0%, which was in accordance with the previous studies conducted on the Chinese population [16,20]. The main result of the study demonstrated that overweight and obese or central obese participants with healthy metabolic status or those with unhealthy metabolic status (regardless of the body fat level) presented a high stroke risk. Compared with these normal-WC and -body-weight individuals, the risk of stroke for MHOW and MHOWC phenotypes significantly increased by 47% and 41%, respectively. MHO exhibited more than a two-fold risk of stroke onset in the second definition of metabolic phenotype. It is worth noting that among metabolically unhealthy individuals, those underweight were at a higher stroke risk than those with normal weight and overweight. We also observed that the stroke risk increased with the increasing number of metabolic abnormalities in different BMI and WC groups.

Various studies have been conducted to explore the relationship between MHO or MHOW phenotype and stroke, and our results are consistent with these studies. A cohort study of 381,363 participants from the UK Biobank reported that MHO was related to an 18% increase in atherosclerotic CVD risk and 10% incident stroke risk with a follow-up of 12.2 years [13]. Similarly, a large-scale cohort study of 458,246 participants from China conducted in five rural and five urban areas demonstrated that MHOW and MHO presented 10% and 11% greater stroke risk, respectively, after a follow-up time of 10 years [16]. The significantly positive associations may partly be explained by the following reasons. Metabolic health is a temporary state, and several studies reported that approximately 20–40% of MHO individuals at baseline might develop abnormal metabolic factors and transition to an unhealthy metabolic status after a follow-up period, leading to a higher risk of stroke [13,16,29,30,31,32]. Atherosclerosis is an essential cause of stroke [33] that may play a critical role in metabolic phenotypes and stroke. A recent analysis of 32,778 Chinese individuals examined the relationship between obese phenotypes and carotid artery plaque (CAP) events and presented a significant positive relationship between MHO and CAP [34]. CAP is a reliable marker of subclinical atherosclerosis, suggesting higher CVD risks [35].

BMI is the most common index used in describing obesity by the assessment of weight and height, but lean and fat mass cannot be discriminated by BMI. In China, the data on the relationship between abdominal obesity phenotype and the risk of stroke are scarce. Therefore, in our analysis, we also defined the metabolic phenotypes based on WC and observed that the results were consistent with the definition based on BMI. It has been demonstrated that central obesity assessed by the WC index was an independent risk factor for cardiometabolic diseases and presented a greater predictive ability for CVD than general obesity assessed by BMI [11,36,37]. Visceral adiposity can lead to metabolic disturbances in adipocyte biology and the pro-inflammatory state in adipose tissue with adverse metabolic consequences, including insulin resistance and alterations in blood pressure regulation and lipid metabolism, which will progress endothelial dysfunction and atherogenesis [38,39]. We used different cut-point values for metabolism and obesity to define phenotypes to enhance the consistency of the results. BP, HbA1c, TG, HDL-C, and LDL-C are significant indicators for identifying hypertension, diabetes, and dyslipidemia. Notably, there is a considerable difference in BP levels (130/85 vs. 130/80 mmHg) and using lower DBP might help identify more individuals with pre-hypertension and with an unhealthy metabolic status. We observed similar positive results in the analyses, supporting the evidence that overweight or obese individuals with a metabolically healthy status were not in a benign condition and presented high stroke risks. In addition, a significant proportion of participants with normal body weight and WC were metabolically unhealthy, presenting an even higher stroke risk. Underweight was reported as a risk factor for stroke [40], and the relatively high stroke risk of MUUW might be attributed to the joint effect of underweight and unhealthy metabolism. People with MUUW phenotype should also be considered a high-risk group for stroke. However, the underlying mechanism of this association requires elucidation in future studies. The prevalence of metabolic syndrome, general obesity, and abdominal obesity has dramatically increased in China [41,42]. Therefore, the early identification of risk factors is extremely important, and effective intervention measures, such as maintaining normal body weight and metabolic health, should be implemented to reduce the burden of stroke.

However, several studies reported that an unhealthy metabolism status rather than obesity was associated with CVD events. A cohort study conducted in Iran reported that all the metabolically unhealthy phenotypes, but not MHO and MHOW, increased CVD risks [19]. A cross-sectional study of 13,239 Chinese adults also reported that MHO did not increase CVD risks [20]. The inconsistency in these results may partly be caused by several reasons. Firstly, the definition and group division of metabolic obesity phenotypes was different in previous studies because there were no uniform criteria for unhealthy metabolism, leading to the prevalence of MHO greatly fluctuating. The cutoff values of general and central obesity vary in different ethnic groups, and the values are lower in Chinese individuals than in Europeans and Americans. Secondly, the sample size and length of follow-up time in previous studies were different, and no increased stroke risk was observed in the relatively shorter follow-up period. In fact, many observational studies report the protective effect of a high BMI on adverse health outcomes, and there is an interesting explanation called the obesity paradox [38,43]. However, “the fatter, the better” should be used cautiously, and further research is urgently required to explore the underlying mechanisms.

In this large-scale prospective cohort study, we examined the stroke risk in metabolic phenotypes grouped by BMI and WC under different definitions. We used the common definition in the articles proposed by Adult Treatment Panel III [26] and an alternative definition adapted from a previous study [13], which can help us better compare our results with those presented in other articles. The outcomes were collected through multi-data linkages across the online information systems. We observed positive relationships, suggesting that the results were unlikely to be due to reverse causality. Previous studies only considered BMI-based metabolic phenotypes and ignored the WC index, which can better reflect the distribution of abdominal fat. To our knowledge, it is the first cohort study among adults in the Chinese community to explore the association of abdominal obesity phenotypes with stroke risk.

Several limitations should be clarified. Firstly, the generalization of the results was limited due to the participants of this study only being from the Songjiang district of Shanghai. Secondly, because the study outcomes were collected from self-report and online information systems rather than active screening, patients with earlier minor strokes who did not seek medical treatment were missed as outcomes and were also included at baseline. Thirdly, in the definition of unhealthy metabolism, only blood pressure, blood glucose, and blood lipid levels were included, and the data on insulin resistance were not collected, which is an essential index that underpins many metabolic disorders [44]. There might be potential misclassification of metabolic status as we considered the drug treatment. People who do not go to the hospital for a prescription but only go to the pharmacy to buy the medications are not recorded in our health information database. Finally, in this study, we concluded that overweight and obese individuals with a healthy metabolism were positively related to high stroke risk. However, we were not able to determine the protective effect of WC reduction and weight loss on stroke. We only considered the obesity and metabolic statuses at baseline, but not the dynamic changes during the follow-up period. Therefore, we need to strengthen the follow-up efforts and explore the impact of dynamic changes in obesity and metabolic statuses on stroke risk in future studies.

## 5. Conclusions

This study determined that the special types of obesity (MHOW/MHO/MHOWC) and all the metabolically unhealthy phenotypes presented positive associations with stroke risks. Individuals who had a normal body weight and WC could be metabolically unhealthy and present even higher stroke risks. We recommend that maintaining normal body weight or WC and improving metabolic health can be of great benefit to prevent and control stroke occurrence and reduce the disease burden of stroke.

## Figures and Tables

**Table 1:** Characteristics of the study participants according to BMI.

Characteristic	All Subjects	BMI				*p* Value
		Underweight (18.5 ≤ BMI < 24.0 kg/m^2^)	Normal Weight (18.5 ≤ BMI < 24.0 kg/m^2^)	Overweight (24.0 ≤ BMI < 28.0 kg/m^2^)	Obesity (BMI ≥ 28.0 kg/m^2^)	
N	34,294	904 (2.64)	15,347 (44.75)	13,488 (39.33)	4555 (13.28)	
Age (years)	56.05 ± 11.26	48.99 ± 15.24	54.69 ± 11.74	57.59 ± 10.06	57.45 ± 10.91	<0.001
Male (%)	13,844 (40.37)	263 (29.09)	5502 (35.85)	6109 (45.29)	1970 (43.25)	<0.001
Education degree (%)						
≤6 years	4871 (14.20)	92 (10.18)	1889 (12.31)	2067 (15.32)	823 (18.07)	<0.001
7–12 years	26,314 (76.73)	561 (62.06)	11,749 (76.56)	10,596 (78.56)	3048 (74.82)	
≥13 years	3109 (9.07)	251 (27.77)	1709 (11.14)	825 (6.12)	324 (7.11)	
Marital status (%)						
Married	31,883 (92.97)	788 (87.17)	14,258 (92.90)	12,621 (93.57)	4216 (92.56)	<0.001
Other ^a^	2411 (7.03)	116 (12.83)	1089 (7.10)	867 (6.43)	339 (7.44)	
Retired (%)	19,783 (57.69)	404 (44.69)	8371 (54.54)	8246 (61.14)	2762 (60.64)	
Place of residence						
Non-urban	19,658 (57.32)	458 (50.66)	8835 (57.57)	7841 (58.13)	2524 (55.41)	<0.001
Urban	14,636 (42.68)	446 (49.34)	6512 (42.43)	5647 (41.87)	2031 (44.59)	
Smoking index, packet year (%)						
None-smoker	26,242 (76.52)	747 (82.63)	12,011 (78.26)	10,037 (74.41)	3447 (75.68)	<0.001
<20.0	2241 (6.53)	51 (5.64)	927 (6.04)	960 (7.12)	303 (6.65)	
20.0–39.9	3160 (9.21)	63 (6.97)	1279 (8.33)	1370 (10.16)	448 (9.84)	
≥40	2651 (7.73)	43 (4.76)	1130 (7.36)	1121 (8.31)	357 (7.84)	
Alcohol drinking (%)						
Never	29,672 (86.52)	833 (92.15)	13,573 (88.44)	11,398 (84.50)	3868 (84.92)	<0.001
Former	359 (1.05)	8 (0.88)	115 (0.75)	170 (1.26)	66 (1.45)	
Current	4263 (12.43)	63 (6.97)	1659 (10.81)	1920 (14.23)	621 (13.63)	
Physical activities (%)						
Low	10,593 (30.89)	378 (41.81)	4767 (31.06)	4024 (29.83)	1424 (31.26)	<0.001
Moderate	21,084 (61.48)	488 (53.98)	9517 (62.01)	8350 (61.91)	2729 (59.91)	
High	2617 (7.63)	38 (4.20)	1063 (6.93)	1114 (8.26)	402 (8.83)	
Fruit intake (g/d)	57.14 (28.57–120.0)	82.86 (28.57–150.0)	71.43 (28.57–142.86)	57.14 (28.57–107.14)	57.14 (28.57–100.0)	<0.001
Vegetable intake (g/d)	242.85 (128.57–400.0)	228.57 (124.05–342.86)	235.15 (128.57–400.0)	242.86 (128.57–401.64)	244.83 (128.58–392.86)	0.026
Fish intake (g/d)	41.97 (20.87–64.29)	41.73 (20.87–63.72)	42.86 (20.87–65.71)	41.73 (20.87–63.72)	41.73 (20.87–63.72)	0.249
Unprocessed meat intake (g/d)	44.50 (27.89–72.44)	46.16 (29.42–78.58)	44.51 (28.57–71.69)	43.97 (27.45–72.25)	43.69 (25.43–73.59)	0.135
Processed meat intake (%)						
Never	17,574 (51.25)	473 (52.32)	7854 (51.18)	7005 (51.94)	2242 (49.22)	0.020
1–3 times/month	13,874 (40.46)	352 (38.94)	6247 (40.71)	5395 (40.0)	1880 (41.27)	
1–3 times/week	2716 (7.92)	76 (8.41)	1188 (7.74)	1033 (7.66)	419 (9.20)	
4–7 times/week	130 (0.38)	3 (0.33)	58 (0.38)	55 (0.41)	14 (0.31)	
BMI (kg/m^2^)	24.38 ± 3.35	17.53 ± 0.81	21.87 ± 1.43	25.75 ± 1.12	30.13 ± 2.13	<0.001
WC (cm)	81.62 ± 9.42	66.01 ± 6.19	75.95 ± 6.78	85.06 ± 6.21	93.62 ± 7.53	<0.001
SBP (mmHg)	133.41 ± 19.38	119.24 ± 16.74	128.99 ± 18.86	136.72 ± 18.50	141.29 ± 18.96	<0.001
DBP (mmHg)	79.99 ± 10.51	73.42 ± 9.68	77.67 ± 10.14	81.67 ± 10.13	84.19 ± 10.50	<0.001
Anti-hypertensive medications (%)	10,863 (31.68)	96 (10.62)	3378 (22.01)	5110 (37.89)	2279 (50.03)	<0.001
TG (mmol/L)	1.34 (0.98–1.92)	0.91 (0.76–1.16)	1.18 (0.88–1.61)	1.50 (1.10–2.12)	1.68 (1.24–2.40)	<0.001
HDL-C (mmol/L)	1.41 ± 0.36	1.69 ± 0.38	1.49 ± 0.35	1.34 ± 0.33	1.28 ± 0.37	<0.001
LDL-C (mmol/L)	2.78 ± 0.83	2.47 ± 0.76	2.75 ± 0.81	2.82 ± 0.85	2.81 ± 0.87	<0.001
Statins (%)	2547 (7.43)	13 (1.44)	720 (4.69)	1212 (8.99)	602 (13.22)	<0.001
FPG (mmol/L)	4.72 (4.26–5.37)	4.56 (4.22–4.92)	4.64 (4.23–5.21)	4.79 (4.28–5.51)	4.92 (4.34–5.75)	<0.001
HbA1c (%)	5.6 (5.3–6.0)	5.4 (5.1–5.7)	5.5 (5.3–5.9)	5.7 (5.4–6.1)	5.8 (5.5–6.3)	<0.001
Metabolic status (%)						
Metabolically healthy	18,759 (54.70)	801 (88.61)	10,508 (68.47)	6028 (44.69)	1422 (31.22)	<0.001
Metabolically unhealthy	15,535 (45.30)	103 (11.39)	4839 (31.53)	7460 (55.31)	3133 (68.78)	
Number of metabolic abnormalities	1.50 ± 1.12	0.56 ± 0.76	1.14 ± 1.01	1.75 ± 1.08	2.13 ± 1.08	<0.001

^a^ Other included unmarried, divorced, separated, and widowed. BMI, body mass index; WC, waist circumference; SBP, systolic blood pressure; DBP, diastolic blood pressure; TG, triglyceride; LDL-C, low-density lipoprotein cholesterol; HDL-C, high-density lipoprotein cholesterol; FPG, fasting plasma glucose; HbA1c, glycated hemoglobin.

**Table 2:** Hazard ratios for stroke according to metabolic phenotypes defined by BMI or WC.

Phenotypes	Cases/Participants	Model 1 ^a^	Model 2 ^b^	Model 3 ^c^	Male ^e^	Female ^e^
		HR (95% CI)	HR (95% CI)	HR (95% CI)	HR (95% CI)	HR (95% CI)
Based on BMI						
MHNW	84/10,508	1.00	1.00	1.00	1.00	1.00
MHUW	5/801	0.78 (0.32, 1.92)	0.99(0.40, 2.44)	1.01 (0.41, 2.49)	0.38 (0.05, 2.76)	1.61 (0.57, 4.56)
MHOW	84/6028	1.76 (1.30, 2.38) ***	1.45 (1.07, 1.97) *	1.47 (1.09, 2.0) *	1.57 (1.07, 2.30) *	1.29 (0.78, 2.12)
MHO	18/1422	1.60 (0.96, 2.67)	1.30 (0.78, 2.16)	1.33 (0.80, 2.22)	1.63 (0.87, 3.06)	0.96 (0.40, 2.29)
MUNW	115/4839	3.06 (2.31, 4.05) ***	2.48 (1.87, 3.29) ***	2.49 (1.87, 3.30) ***	2.18 (1.48, 3.23) ***	2.73 (1.79, 4.15) ***
MUUW	4/103	5.15 (1.89, 14.04) **	4.23 (1.55, 11.54) **	3.92 (1.44, 10.72) **	4.61 (1.11, 19.07) *	3.38 (0.81, 14.07)
MUOW	158/7460	2.73 (2.10, 3.56) ***	2.10 (1.61, 2.73) ***	2.14 (1.64, 2.79) ***	2.16 (1.53, 3.06) ***	2.01 (1.32, 3.06) **
MUO	78/3133	3.22 (2.37, 4.38) ***	2.56 (1.88, 3.49) ***	2.60 (1.91, 3.55) ***	2.45 (1.61, 3.72) ***	2.65 (1.66, 4.23) ***
Based on WC ^d^						
MHNWC	114/13,160	1.00	1.00	1.00	1.00	1.00
MHOWC	77/5599	1.60 (1.20, 2.13) **	1.43 (1.06, 1.92) *	1.41 (1.02, 1.94) *	1.31 (0.85, 2.02)	1.67 (1.01, 2.76) *
MUNWC	167/7129	2.79 (2.20, 3.54) ***	2.21 (1.74, 2.81) ***	2.25 (1.76, 2.87) ***	1.94 (1.46, 2.58) ***	2.86 (1.81, 4.53) ***
MUOWC	188/8406	2.65 (2.09, 3.34) ***	2.23 (1.76, 2.84) ***	2.16 (1.63, 2.87) ***	1.65 (1.14, 2.39) **	2.94 (1.86, 4.66) ***

BMI, body mass index; WC, waist circumference; MHNW, Metabolically healthy and normal weight; MHUW, Metabolically healthy and underweight; MHOW, Metabolically healthy and overweight; MHO, Metabolically healthy and obese; MUNW, Metabolically unhealthy and normal weight; MUUW, Metabolically unhealthy and underweight; MUOW, Metabolically unhealthy and overweight; MUO, Metabolically unhealthy and obese; MHNWC, Metabolically healthy and normal WC; MHOWC, Metabolically healthy and oversized WC; MUNWC, Metabolically unhealthy and normal WC; MUOWC, Metabolically unhealthy and oversized WC; ^a^ Model 1, unadjusted. ^b^ Model 2, adjusted for gender and age. ^c^ Model 3, adjusted for gender, age, education degree, marital status, retirement, place of residence, smoking index, alcohol drinking, physical activities, and consumption of fruit, vegetables, fish, and processed and unprocessed meats. ^d^ BMI was additionally adjusted in Model 3. ^e^ Adjusted for the same variables as Model 3. * *p* < 0.05, ** *p* < 0.01, *** *p* < 0.001.

**Table 3:** Association between the number of metabolic abnormalities and risk of stroke in different BMI and WC groups.

Variables	Number of Metabolic Abnormalities					*p* for Trend
	0	1	2	3	4	
BMI group (kg/m^2^)						
<24.0	1.00	1.74 (1.05, 2.88)	3.05 (1.84, 5.06)	4.77 (2.75, 8.28)	6.88 (3.49, 13.59)	<0.001
≥24.0	1.00	1.71 (0.91, 3.19)	2.29 (1.23, 4.25)	2.66 (1.42, 5.01)	3.05 (1.55, 6.02)	<0.001
WC group ^a^						
Normal WC	1.00	1.58 (1.00, 2.50)	2.77 (1.75, 4.38)	3.46 (2.09, 5.74)	5.65 (3.10, 10.29)	<0.001
Oversized WC	1.00	2.28 (1.04, 4.98)	2.82 (1.30, 6.12)	3.63 (1.66, 7.94)	3.53 (1.53, 8.12)	<0.001

BMI, body mass index; WC, Waist circumference; The model was fully adjusted for gender, age, education degree, marital status, retirement, place of residence, smoking index, alcohol drinking, physical activities, and consumption of fruit, vegetables, fish, and processed and unprocessed meats; ^a^ BMI was additionally adjusted in the model.

## Data Availability

The data sets used and analyzed in this study are available from the corresponding author upon reasonable request.

## Supplementary Materials

The following supporting information can be downloaded at: https://www.mdpi.com/article/10.3390/nu14245258/s1, Table S1: Characteristics of the study participants according to WC.

## Abbreviations

BMI	Body mass index
WC	Waist circumference
MHNW	Metabolically healthy and normal weight
MHUW	Metabolically healthy and underweight
MHOW	Metabolically healthy and overweight
MHO	Metabolically healthy and obese
MUNW	Metabolically unhealthy and normal weight
MUUW	Metabolically unhealthy and underweight
MUOW	Metabolically unhealthy and overweight
MUO	Metabolically unhealthy and obese
MHNWC	Metabolically healthy and normal WC
MHOWC	Metabolically healthy and oversized WC
MUNWC	Metabolically unhealthy and normal WC
MUOWC	Metabolically unhealthy and oversized WC
CVD	Cardiovascular disease
SSACB	Shanghai Suburban Adult Cohort and Biobank
MET	Metabolic equivalent task
SBP	Systolic blood pressure
DBP	Diastolic blood pressure
TG	Triglyceride
LDL-C	Low-density lipoprotein cholesterol
HDL-C	High-density lipoprotein cholesterol
FPG	Fasting plasma glucose
HbA1c	Glycated hemoglobin
EMR	Electronic Medical Record
WHO	World Health Organization
ICD-10	10th Revision of the International Classification of Diseases
SD	Standard deviation
HR	Hazard ratio
CI	Confidence interval
CAP	Carotid artery plaque
