# Supplementary material for: Association between Metabolic Phenotypes of Body Fatness and Incident Stroke: A Prospective Cohort Study of Chinese Community Residents
Source: Nutrients. 2022 Dec 9;14(24):5258. doi: 10.3390/nu14245258 (PMC9785835; doi:10.3390/nu14245258)
Supplement: Supplementary file 1 [file nutrients-14-05258-s001.zip › nutrients-2051536-supplementary.pdf]

nutrients

MDPI

Supplementary Materials

Association between Metabolic Phenotypes of Body Fatness and Incident Stroke: A Prospective Cohort Study of Chinese Community Residents

Minhua Tang [1,2], Qi Zhao [1], Kangqi Yi [1], Yiling Wu [2], Yu Xiang [1], Maryam Zaid [1], Shuheng Cui [1], Xuyan Su [2], Yuting Yu [1], Genming Zhao [1,*] and Yonggen Jiang [2,*]

Table S1. Characteristics of the study participants according to WC.

Characteristic	All subjects	WC: Normal (WC < 90 cm for men and < 80 cm for women)	WC: Oversized (WC ≥ 90 cm for men and ≥ 80 cm for women)	*p* Value
N	34,294	20,289 (59.16)	14,005 (40.84)	
Age (years)	56.05 ± 11.26	54.26 ± 11.82	58.63 ± 9.84	< 0.001
Male (%)	13,844 (40.37)	9869 (48.64)	3975 (28.38)	< 0.001
Education degree (%)				
≤ 6 years	4871 (14.20)	2031 (10.01)	2840 (20.28)	< 0.001
7–12 years	26,314 (76.73)	15,827 (78.01)	10,487 (74.88)	
≥ 13 years	3109 (9.07)	2431 (11.98)	678 (4.84)	
Marital status (%)				
Married	31,883 (92.97)	18,941 (93.36)	12,942 (92.41)	< 0.001
Other [a]	2411 (7.03)	1348 (6.64)	1063 (7.59)	
Retired (%)	19,783 (57.69)	10379 (51.16)	9404 (67.15)	< 0.001
Place of residence				
Non-urban	19,658 (57.32)	11,158 (55.0)	8500 (60.69)	< 0.001
Urban	14,636 (42.68)	9131(45.0)	5505 (39.31)	
Smoking index, packet year (%)				
None-smoker	26,242 (76.52)	14,556 (71.74)	11,686 (83.44)	< 0.001
< 20.0	2241 (6.53)	1652 (8.14)	589 (4.21)	
20.0–39.9	3160 (9.21)	2227 (10.98)	933 (6.66)	
≥ 40	2651 (7.73)	1854 (9.14)	797 (5.69)	
Alcohol drinking (%)				
Never	29,672 (86.52)	17,146 (84.51)	12,526 (89.44)	< 0.001
Former	359 (1.05)	225 (1.11)	134 (0.96)	
Current	4263 (12.43)	2918 (14.38)	1345 (9.60)	
Physical activities (%)				
Low	10,593 (30.89)	7034 (34.67)	3559 (25.41)	< 0.001
Moderate	21,084 (61.48)	11,941 (58.85)	9143 (65.28)	
High	2617 (7.63)	1314 (6.48)	1303 (9.30)	
Fruit intake (g/d)	57.14 (28.57–120.0)	71.43 (28.57–142.86)	57.14 (28.57–100.0)	< 0.001
Vegetable intake (g/d)	242.85 (128.57–400.0)	242.86 (128.58–403.29)	235.15 (120.87–371.43)	< 0.001
Fish intake (g/d)	41.97 (20.87–64.29)	42.87 (22.19–71.43)	41.72 (19.74–63.72)	< 0.001
Unprocessed meat intake (g/d)	44.50 (27.89–72.44)	46.81 (29.09–78.01)	42.55 (24.16–68.24)	< 0.001
Processed meat intake (%)				

Never	17,574 (51.25)	10,337 (50.95)	7237 (51.67)	0.104
1–3 times/month	13,874 (40.46)	8216 (40.49)	5658 (40.40)	
1–3 times/week	2716 (7.92)	1663 (8.20)	1053 (7.52)	
4–7 times/week	130 (0.38)	73 (0.36)	57 (0.41)	
BMI (kg/m^2)	24.38 ± 3.35	22.77 ± 2.55	26.71 ± 2.96	< 0.001
WC (cm)	81.62 ± 9.42	76.28 ± 7.0	89.36 ± 6.72	< 0.001
SBP (mmHg)	133.41 ± 19.38	129.79 ± 18.89	138.66 ± 18.87	< 0.001
DBP (mmHg)	79.99 ± 10.51	78.81 ±10.50	81.71 ± 10.29	< 0.001
Anti-hypertensive medications (%)	10,863 (31.68)	4947 (24.38)	5919 (42.24)	< 0.001
TG (mmol/L)	1.34 (0.98–1.92)	1.22 (0.90–1.71)	1.55 (1.14–2.19)	< 0.001
HDL-C (mmol/L)	1.41 ± 0.36	1.45 ± 0.36	1.35 ± 0.35	< 0.001
LDL-C (mmol/L)	2.78 ± 0.83	2.73 ± 0.80	2.86 ± 0.87	< 0.001
Statins (%)	2547 (7.43)	968 (4.77)	1579 (11.27)	< 0.001
FPG (mmol/L)	4.72 (4.26–5.37)	4.69 (4.25–5.26)	4.79 (4.27–5.57)	< 0.001
HbA1c (%)	5.6 (5.3–6.0)	5.6 (5.3–5.9)	5.8 (5.4–6.15)	< 0.001
Metabolic status (%)				
Metabolically healthy	18,759 (54.70)	13,160 (64.86)	5599 (39.98)	< 0.001
Metabolically unhealthy	15,535 (45.30)	7129 (35.14)	8406 (60.02)	
Number of metabolic abnormalities	1.50 ± 1.12	1.23 ± 1.04	1.88 ± 1.10	< 0.001

[a] Other included unmarried, divorced, separated, and widowed. WC, waist circumference; SBP, systolic blood pressure; DBP, diastolic blood pressure; TG, triglyceride; LDL-C, low-density lipoprotein cholesterol; HDL-C, high-density lipoprotein cholesterol; FPG, fasting plasma glucose; HbA1c, glycated hemoglobin.
